# Ratiometric Optical Temperature Sensor Using Two Fluorescent Dyes Dissolved in an Ionic Liquid Encapsulated by Parylene Film

**DOI:** 10.3390/s130404138

**Published:** 2013-03-27

**Authors:** Tetsuo Kan, Hironori Aoki, Nguyen Binh-Khiem, Kiyoshi Matsumoto, Isao Shimoyama

**Affiliations:** 1 Department of Mechano-Informatics, Graduate School of Information Science and Technology, The University of Tokyo, 7-3-1 Hongo, Bunkyo-ku, 113-8656 Tokyo, Japan; E-Mails: g640608@gmail.com (H.A.); isao@i.u-tokyo.ac.jp (I.S.); 2 IRT Research Initiative, The University of Tokyo, 7-3-1 Hongo, Bunkyo-ku, 113-8656 Tokyo, Japan; E-Mails: khiem@leopard.t.u-tokyo.ac.jp (N.B.-K.); matsu@leopard.t.u-tokyo.ac.jp (K.M.)

**Keywords:** temperature sensor, ionic liquid, fluorescent dye, Parylene on liquid deposition (PoLD), 07.20.Dt

## Abstract

A temperature sensor that uses temperature-sensitive fluorescent dyes is developed. The droplet sensor has a diameter of 40 μm and uses 1 g/L of Rhodamine B (RhB) and 0.5 g/L of Rhodamine 110 (Rh110), which are fluorescent dyes that are dissolved in an ionic liquid (1-ethyl-3-methylimidazolium ethyl sulfate) to function as temperature indicators. This ionic liquid is encapsulated using vacuum Parylene film deposition (which is known as the Parylene-on-liquid-deposition (PoLD) method). The droplet is sealed by the chemically stable and impermeable Parylene film, which prevents the dye from interacting with the molecules in the solution and keeps the volume and concentration of the fluorescent material fixed. The two fluorescent dyes enable the temperature to be measured ratiometrically such that the droplet sensor can be used in various applications, such as the wireless temperature measurement of microregions. The sensor can measure the temperature of such microregions with an accuracy of 1.9 °C, a precision of 3.7 °C, and a fluorescence intensity change sensitivity of 1.0%/K. The sensor can measure temperatures at different sensor depths in water, ranging from 0 to 850 μm. The droplet sensor is fabricated using microelectromechanical system (MEMS) technology and is highly applicable to lab-on-a-chip devices.

## 1. Introduction

The temperature control of microfluidic channels is essential for lab-on-a-chip experiments, such as capillary electrophoresis [1]. The temperature in these microfluidic channels is typically measured by imaging temperature-sensitive materials dissolved in liquids. This method enables the wireless sensing of remote temperatures and is thus highly applicable to measuring the temperatures of small fluidic channels. Fluorescent dyes (or thermochromic liquid crystals), and more recently quantum dots, have been used as temperature-sensitive materials because their photoemission intensities are temperature dependent [2– 8]. This method is versatile, but also possesses several drawbacks. First, the method is sensitive to changes in the material concentration, which causes fluctuations in the photoemission intensity, resulting in inaccurate temperature measurements. Second, the material typically becomes contaminated in the solution, which can hinder the chemical reaction in the microchannel. Thus, it is desirable to develop a method in which the material does not interact with the chemicals in the solution.

In this paper, we develop a temperature sensor using fluorescent dye droplets that are encapsulated by an impermeable polymer thin film. Our group previously developed a method to vacuum seal nonvolatile liquids using a Parylene coating, which is known as PoLD (Parylene on liquid deposition) [9,10]. Two fluorescent dyes were dissolved in a nonvolatile ionic liquid to enable ratiometric temperature measurements. The droplets were patterned on a substrate and then encapsulated by a Parylene film, which fixed the volume and concentration of the droplets. The dye could not interact with the chemicals in the solution because the droplets were sealed by the chemically stable film.

## 2. Sensor Configurations and Sensing Method

**Figure 1.**
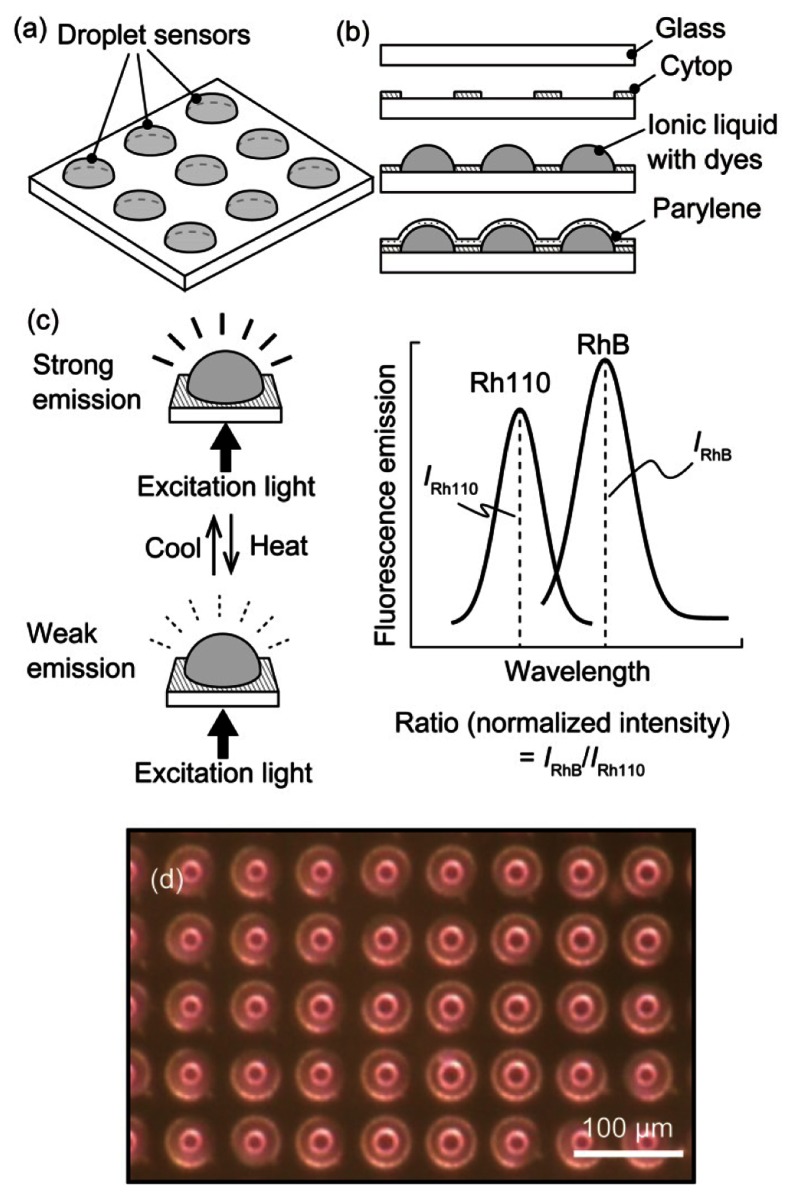
(**a**) Schematic of the droplet sensor, (**b**) fabrication process of the droplet sensor, (**c**) temperature measurement method using fluorescence intensity, and (**d**) image of fabricated droplet sensors.

In the developed sensor, the liquid in which the temperature sensitive dyes are dissolved was coated with a Parylene thin film (Figure 1(a)). The quantum yield *Φ* of a fluorescent dye is temperature dependent; therefore, a temperature change modulated the fluorescence intensity [11], which decreased with increasing temperature (see Figure 1(c)). We used a ratiometric method to determine the temperature from the fluorescence intensity using two dyes, Rhodamine B (RhB) and Rhodamine 110 (Rh110) [12]. The fluorescence of these two dyes could be measured independently because the dyes have different excitation/emission wavelengths. The temperature could also be measured ratiometrically because the dyes exhibited different thermal dependences. The ratiometric method is robust to artifacts from optical losses by the absorption of medium because any optical loss is cancelled out during the ratiometric operation. These dyes were activated as fluorescent materials by dissolution in an ionic liquid; we previously confirmed that the dyes behaved similarly in the ionic liquid as in water. The ionic liquid had a very low vapor pressure, was nonvolatile, and could be encapsulated using chemical vapor deposition, as we reported previously [9,10]. The Parylene-C film coating prevented liquid leakage, enabling the application of the sensor to aqueous environments.

## 3. Device Fabrication and Experimental Apparatus

The droplet sensor was fabricated using microelectromechanical system (MEMS) microfabrication technology. The droplets were patterned on a glass substrate (see Figure 1(b)). First, a Cytop (Asahi Glass, Tokyo, Japan) hydrophobic layer was coated onto a cover glass. The glass was spun at 3,000 rpm for 20 s, followed by sequential baking at 80 °C for 30 min and at 180 °C for 30 min. Next, a thin layer of aluminum (Al) was deposited to act as an etching mask, *i.e.*, the Al layer was patterned to create circular openings in the Cytop film. The circular openings were produced via oxygen (O_2_) plasma etching of the Cytop layer. Ionic liquids adhere to hydrophilic surfaces (in this case, glass); therefore, the opening determined the outer shape of the liquid. In this paper, a diameter of 40 μm was adopted for the circular opening. The 1-ethyl-3-methylimidazolium ethyl sulfate ionic liquid containing the dissolved RhB and Rh110 fluorescent dyes was manually dropped onto the openings. In the subsequent experiments, the concentrations of the dyes were maintained at 1 g/L (RhB) and 0.5 g/L (Rh110). The droplets were then encapsulated with Parylene C via chemical vapor deposition (CVD) (using a monomer weight of 0.5 g, which corresponded to a 1-μm-thick Parylene layer). The Parylene C was directly deposited onto the liquid surface to form a polymer film because the ionic liquid does not evaporate, even in a vacuum chamber. In the experiments described below, the droplet sensor outputs were compared with the temperature measured by a conventional thermocouple. A thermocouple was placed near the droplet sensor by embedding the thermocouple in a polydimethylsiloxane (PDMS) (Sylgard 184, Dow Corning, Midland, MI, USA) elastomer with the fabricated droplets. A thermocouple was also used for the temperature feedback control in the experiments. Figure 1(d) shows an image of a fabricated array of highly uniform encapsulated droplets.

The experimental apparatus consisted of an inverted fluorescent microscope (Axiovert 200, Carl Zeiss Group, Oberkochen, Germany). The sensor substrate was illuminated by a bandpass-filtered mercury (Hg) lamp. The emission light was collected with an objective lens (LUCPL FLN ×40, N.A. 0.6, Olympus Corporation, Tokyo, Japan) and measured using a charge-coupled device (CCD) camera (ORCA-ER, Hamamatsu, Shizuoka, Japan). The filter sets were changed manually to measure the dye fluorescence. A silicon rubber heater was attached to the top of the sensor such that the heater did not interfere with the optical path. The temperature of the sensor was controlled by a thermo-control unit (E5BS, OMRON, Kyoto, Japan) using the output of the embedded thermocouple.

## 4. Experiments and Results

### 4.1. Data Collection Method

**Figure 2.**
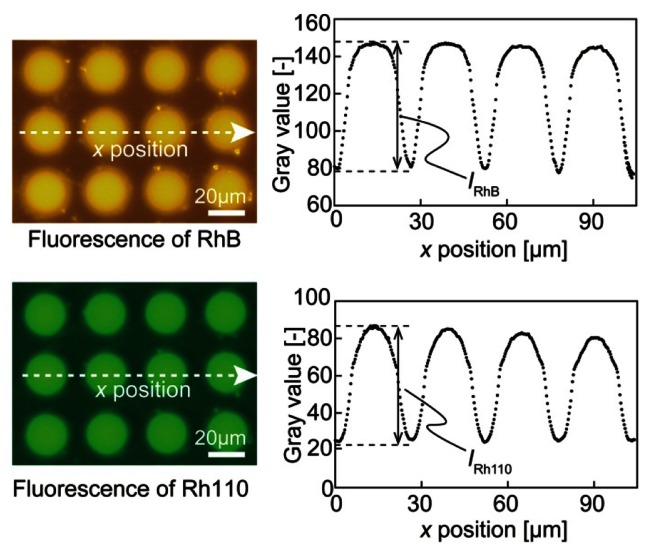
Data acquisition scheme.

The peak wavelengths of the emission spectrum of RhB and Rh110 have been reported to be 592 nm and 538 nm, respectively. For RhB, an excitation filter passing wavelengths from 550 to 580 nm and an emission filter passing wavelengths from 590 nm to 650 nm were used. For Rh110, an excitation filter passing wavelengths from 475 to 495 nm and an emission filter passing wavelengths from 515 nm to 565 nm were used. There was little overlap between the excitation and emission spectra of the two dyes; therefore, there was negligible incorporation of fluorescence between the two dyes. Thus, the experimental data were analyzed assuming that the fluorescence of the two dyes was completely separable. Figure 2 presents color fluorescence images for each dye and the intensity distribution from the CCD plots. The wafer surface had a background fluorescence level in the absence of droplets. The sensor fluorescence intensity was defined as the difference between the maximum and minimum gray values, which weredenoted by *I*_RhB_ for RhB and *I*_Rh110_ for Rh110 (see Figure 2). The background fluorescence level was extracted from the experimental data.

### 4.2. Sensor Characteristics: Photobleaching and Dispersion of Droplets

**Figure 3.**
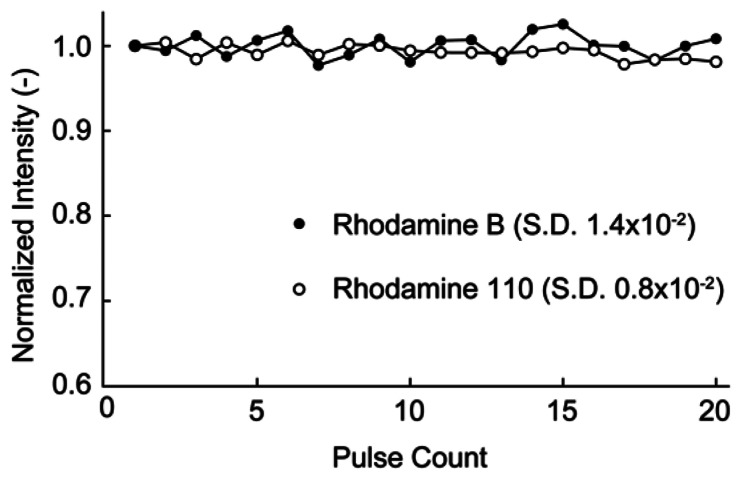
Plot showing stable dye intensities over multiple pulse excitations.

**Figure 4.**
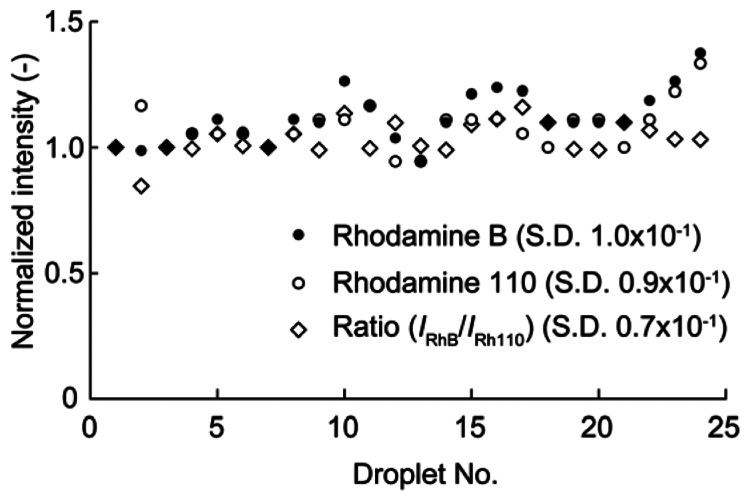
Variation in the fluorescence intensity of the sensor with the droplet number.

Fluorescent dyes may be photobleached by continuous illumination by exciting light. To inhibit the photobleaching effect, pulse illuminations were used for the droplet sensor. The duration of a single pulse was 0.75 s, which was adequate for the temperature measurements. Figure 3 shows the fluorescence intensity of each dye *versus* the pulse count. The fluorescence intensity was normalized by the initial intensity for each dye. The fluorescence intensities of RhB and Rh110 after 20 pulses were 101% and 98% of the initial dye intensities, respectively. Thus, almost no photobleaching occurred using pulse excitation for several tens of pulses. Consequently, pulse excitation was used in the temperature measurements. The uniformity of the fabricated temperature sensors was also evaluated. The fluorescence intensities of RhB and Rh110 were measured for 25 droplets, and the ratio of the two intensities was calculated for each droplet (see Figure 4). The intensity of each droplet was normalized by that of the first droplet, which was selected arbitrarily. Although the individual droplet intensities of the RhB and Rh110 exhibited relatively large fluctuations of up to 30% of the initial droplet intensity, the variation in the ratio of the RhB and Rh110 intensities was as small as 6%.

### 4.3. Measurement of the Liquid Temperature

**Figure 5.**
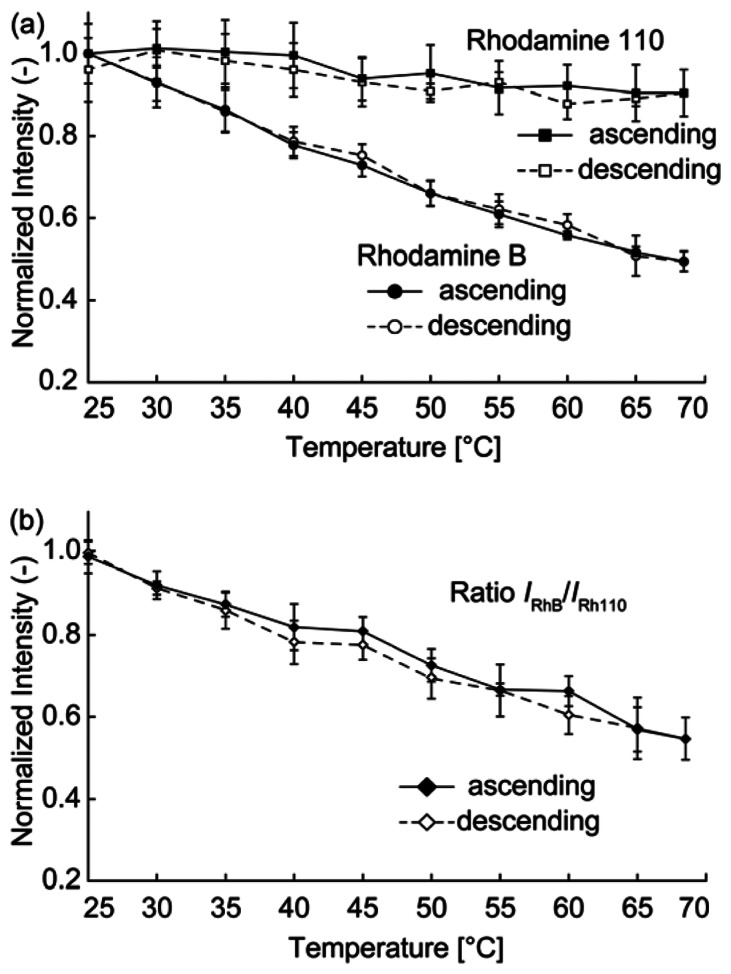
Sensor response to temperature changes: (**a**) measurements for individual dyes and (**b**) ratiometric measurement.

The relationship between the temperature and fluorescence intensity was investigated for each dye. The temperature was varied by heating the sensor with a silicon rubber heater. The sensor was heated from 25 °C to 68 °C (and then cooled from 68 °C to 25 °C) using the heater, and the fluorescence intensity of the droplet was simultaneously measured. Figure 5(a) shows plots of the intensities of each dye. The fluorescence intensity of each dye was normalized by its fluorescence intensity at 25 °C. The fluorescence intensity for both dyes decreased with increasing temperature. We performed intensity measurements in ascending steps (increasing temperature) and descending steps (decreasing temperature). The hysteresis was sufficiently small that it was neglected. A linear fit to the temperature data was obtained using the least-mean-squares method, resulting in temperature dependences of the RhB and Rh110 intensities of −1.2%/K and −0.27%/K, respectively. Figure 5(b) shows a plot of the *I*_RhB_/*I*_Rh110_ ratio, where *I*_RhB_ and *I*_Rh110_ denote the fluorescence intensities of RhB and Rh110, respectively. The temperature dependency of the ratio was −1.0%/K. Assuming that the fits were accurate, the sensor accuracy was defined as the difference between the measured fluorescence intensity and the fluorescence intensity calculated from the linear fit. A mean accuracy of 1.9 °C was obtained by calculating the accuracy of each measured point in the ascending direction in Figure 5(b). The temperature precision was determined to be 3.7 °C, which corresponded to the largest standard deviation of the sensor output (*i.e.*, the error bars) observed in the ratiometric data.

**Figure 6.**
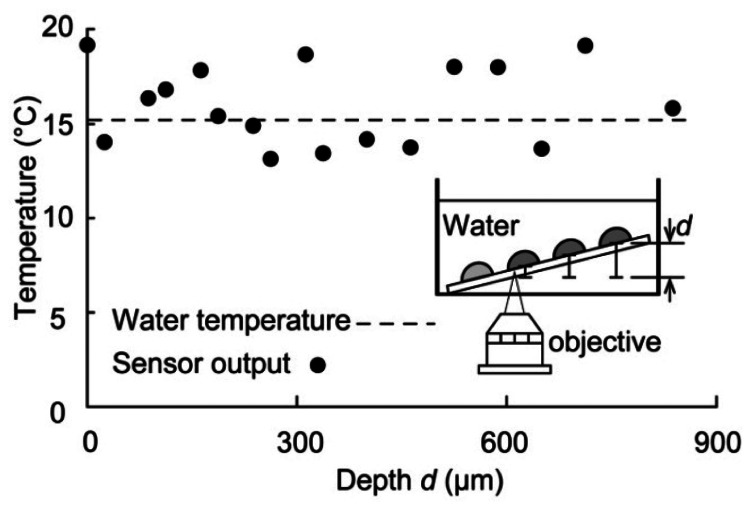
Variation in sensor output for different optical path lengths.

Finally, the temperature was measured when the sensor was immersed in water to determine whether the sensor could function effectively in water and to measure a constant temperature value independent of the optical path between the droplet and objective lens. The inset in Figure 6 shows the experimental apparatus. The water temperature was held constant, and the sensor was inclined at an angle such that the water depth (and thus the optical path) was different for each droplet. The temperature determined from the fluorescence intensity of the droplet sensor was compared with the reference temperature measured with a thermocouple. The intensities of 17 sensor droplets were measured at different depths *d*, ranging from 0 μm (initial depth) to approximately 850 μm (see the inset of Figure 6). The water temperature was 15 °C, and the measured temperature at different depths was distributed around approximately 15 °C. Although there were some errors in the measured temperature, the sensor precision was 3.7 °C (the temperature precision of the droplet sensor) over the entire depth. Therefore, the temperature was not affected by the sensor depth. Our sensor can thus be used for temperature measurements that involve variations in the optical path between the objective lens and droplet.

## 5. Conclusions

We have developed a microdroplet temperature sensor that is encapsulated using the PoLD method. The droplet sensor has a diameter of 40 μm and uses Rhodamine B and Rhodamine 110 fluorescent dyes dissolved in an ionic liquid (1-ethyl-3-methylimidazolium ethyl sulfate) as temperature indicators. The droplet sensor measures the temperature using the ratiometric fluorescence intensity with an accuracy of 1.9 °C, a precision of 3.7 °C, and a sensitivity of 1.0%/K. In addition, the temperature can be measured for varying sensor depths in water, ranging from 0 to 850 μm. The concentration of the fluorescent dyes and sensor volume are fixed by the PoLD encapsulation, which prevents the chemicals in the solution in the microchannel from interacting w ith the temperature indicator dyes. The reported encapsulation was verified to be sufficiently stable in a water environment for 40 min [13]. In addition, the fluorescent dye Rhodamine B has been reported to show no photobleaching after 30 min of exposure [5]. The sensor can thus be applied for measurements lasting as long as 30 min. The sensitivity and accuracy of the temperature measurement can be improved by optimizing the dye concentrations and by identifying optimal fluorescent dyes. The droplet position and size can be controlled using MEMS technology, making this sensor highly applicable to micro total analysis systems (μ-TASs).
